# Frequent *DPH3* promoter mutations in skin cancers

**DOI:** 10.18632/oncotarget.5771

**Published:** 2015-09-21

**Authors:** Evgeniya Denisova, Barbara Heidenreich, Eduardo Nagore, P. Sivaramakrishna Rachakonda, Ismail Hosen, Ivana Akrap, Víctor Traves, Zaida García-Casado, José Antonio López-Guerrero, Celia Requena, Onofre Sanmartin, Carlos Serra-Guillén, Beatriz Llombart, Carlos Guillén, Jose Ferrando, Enrique Gimeno, Alfred Nordheim, Kari Hemminki, Rajiv Kumar

**Affiliations:** ^1^ Division of Molecular Genetic Epidemiology, German Cancer Research Center, Heidelberg, Germany; ^2^ Department of Dermatology, Instituto Valenciano de Oncologia, Valencia, Spain; ^3^ Interfaculty Institute of Cell Biology, Tuebingen University, and IMPRS (“From Molecules to Organisms”), Tuebingen, Germany; ^4^ Department of Pathology, Instituto Valenciano de Oncologia, Valencia, Spain; ^5^ Laboratory of Molecular Biology, Instituto Valenciano de Oncologia, Valencia, Spain; ^6^ Departments of Pathology & Dermatology, Hospital Arnau de Vilanova, Valencia, Spain; ^7^ German Cancer Consortium (DKTK/DKFZ) Heidelberg, Germany; ^8^ Center for Primary Health Care Research, Lund University, Malmö, Sweden

**Keywords:** DPH3, OXNAD1, whole-exome sequencing, noncoding mutations, skin cancers

## Abstract

Recent reports suggested frequent occurrence of cancer associated somatic mutations within regulatory elements of the genome. Based on initial exome sequencing of 21 melanomas, we report frequent somatic mutations in skin cancers in a bidirectional promoter of *diphthamide biosynthesis 3* (*DPH3*) and *oxidoreductase NAD-binding domain containing 1* (*OXNAD1*) genes. The UV-signature mutations occurred at sites adjacent and within a binding motif for E-twenty six/ternary complex factors (Ets/TCF), at −8 and −9 bp from DPH3 transcription start site. Follow up screening of 586 different skin lesions showed that the DPH3 promoter mutations were present in melanocytic nevi (2/114; 2%), melanoma (30/304; 10%), basal cell carcinoma of skin (BCC; 57/137; 42%) and squamous cell carcinoma of skin (SCC; 12/31; 39%). Reporter assays carried out in one melanoma cell line for *DPH3* and *OXNAD1* orientations showed statistically significant increased promoter activity due to −8/−9CC > TT tandem mutations; although, no effect of the mutations on *DPH3* and *OXNAD1* transcription in tumors was observed. The results from this study show occurrence of frequent somatic non-coding mutations adjacent to a pre-existing binding site for Ets transcription factors within the directional promoter of *DPH3* and *OXNAD1* genes in three major skin cancers. The detected mutations displayed typical UV signature; however, the functionality of the mutations remains to be determined.

## INTRODUCTION

Emerging reports have identified frequent somatic mutations within regulatory sequences of the human genome [1-4]. Initially, the only regulatory mutations that are common in many cancers were reported in the core promoter of the telomerase reverse transcriptase (*TERT)* gene, which increase *TERT* transcription through creation of binding motifs for E-twenty six/ternary complex (Ets/TCF) transcription factors [1, 2, 5, 6]. Recent initiatives directed at genome wide search for non-coding regulatory mutations have uncovered alterations upstream of a number of genes [3, 4, 7]. In particular, the non-coding mutations in the *succinate dehydrogenase complex, subunit D, integral membrane protein (SDHD)* and *diphthamide biosynthesis 3 (DPH3)* promoters have been shown to affect Ets binding motifs and occurred in melanoma at frequencies of 4-10 and 13%, respectively [3, 4, 8]. The non-coding mutations add to the complexity of melanoma genome, which is characterized by one of the highest prevalence of somatic mutations [9-18].

In this report we show that typical ultraviolet (UV) signature mutations in the promoter region of *DPH3* and oxidoreductase NAD-binding domain containing 1 (*OXNAD1)* genes (henceforth, called *DPH3* promoter mutations), adjacent and within a preexisting Ets binding site, occur not only in melanoma but are common in basal cell (BCC) and squamous cell (SCC) carcinomas of skin. The mutations in the *DPH3* promoter putatively abrogate the pre-existing binding motif for Ets transcription factors, whereas the mutations in *TERT* promoter result in de novo creation of those motifs.

## RESULTS

Mutations in the *DPH3* promoter were initially detected by exome-sequencing of 21 paired DNA from primary tumors and corresponding blood tissues from melanoma patients (Suppl. Table S1). Sequencing reads supporting C > T mutations at hg19 coordinate chr3:16,306,504 (8 bp upstream of *DPH3* RefSeq transcription start site (TSS) - hence named −8C > T - and 163 bp upstream of *OXNAD1* RefSeq TSS) and/or chr3:16,306,505 (9 bp upstream of *DPH3* RefSeq TSS - hence named −9C > T - and 162 bp upstream of *OXNAD1* RefSeq TSS) were discovered in 6 tumors with allelic fraction ranging from 1.4 to 32% (Suppl. Table S2). One C > T mutation at −9 bp position and one CC > TT tandem mutation at −8/−9 bp positions with allelic fractions of 32% and 20%, respectively, were confirmed by Sanger sequencing (Suppl. Figure S1).

**Figure 1:**
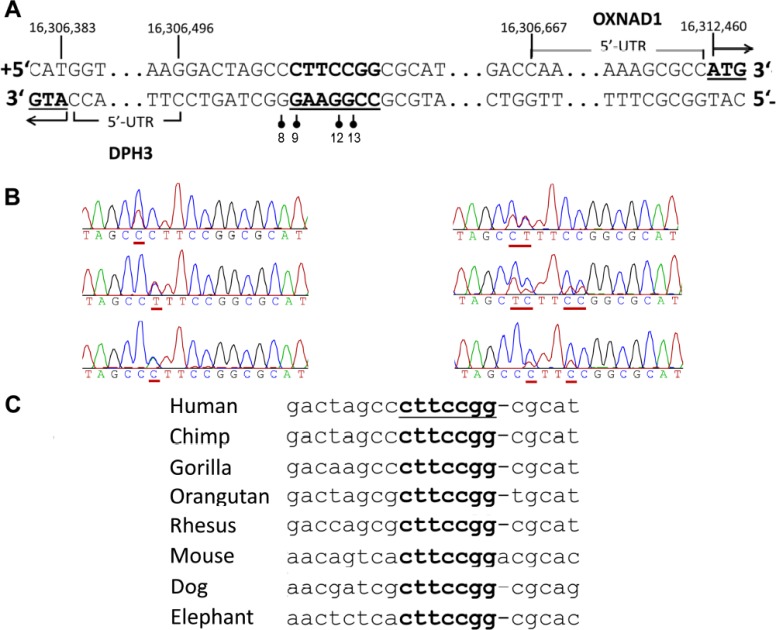
Recurrent somatic mutations in the *DPH3* promoter region **A.** The mutated positions (indicated with vertical bars with filled circles) in the promoter region of the *DPH3* gene adjacent and within a predicted Ets/TCF binding site, which is shown in bold and underlined. Positions “8”, “9”, “12” and “13” correspond to the distance from transcription start site (TSS; RefSeq) of *DPH3*, which is transcribed from negative strand. Those mutated sites correspond to 163, 162, 159 and 158 bp positions from *OXNAD1* TSS (RefSeq), respectively, which is transcribed from positive strand in opposite orientation. **B.** Representative Sanger sequencing chromatograms with mutated positions underlined. **C.** Multiple alignment of the mutated region from different species (UCSC genome browser, Multiz Alignments of 100 Vertebrates). The positions of mutations at “9”, “12” and “13” bp are conserved, the conserved motif is underlined.

Follow up sequencing of 304 melanomas showed that those mutations, lying within and adjacent to a preexisting Ets/TCF binding motif CCTTCCGG (CCGGAAGG on the reverse strand were present in 30 (10%) tumors (Table 1; Figure 1A, 1B).

The most recurrent mutations were C > T transitions at −8 (14; 5%) and −9 bp (12; 4%) followed by −8/−9CC > TT tandem mutations (2; 1%; Figure 1B). Other mutations included one C > A transversion at −9 bp and one C > T transition at −12 bp. The C > T mutation at −12 bp in two melanomas co-occurred with −8C > T and −9C > T mutations, respectively (Table 1). The *DPH3* promoter mutations were also detected in two (one −8C > T and one −9C > T) of the 114 melanocytic nevi. In addition the screening showed that the mutations were present in 57 of 137 (42%) BCC and in 12 out of 31 (39%) SCC (Table 1). We also screened DNA from skin tissues surrounding tumors from 119 BCC and 19 SCC patients and did not detect any *DPH3* promoter mutations.

**Table 1:** Frequency of *DPH3* promoter mutations in different cancer types

	Melanoma n=304	Basal Cell Carcinoma n=137^c^	Squamous Cell Carcinoma n=31^e^
All mutations	30 (10%)	57 (42%)	12 (39%)
−8C>T	14^a^ (5%)	27 (20%)	3 (9%)
−9C>T	12^b^ (4%)	13 (9%)	5 (16%)
−8/−9CC>TT	2 (1%)	14^d^ (10%)	3^f^ (10%)
−9C>A	1	2	1
−12C>T	1	1	0

Mutation names refer to the position from *DPH3* TSS (RefSeq) and correspond to the following hg19 coordinates: −8C>T = chr3:16,306,504 C>T mutations, −9C>T/A = chr3:16,306,505 C>T/A mutations, −8/9CC>TT = chr3:16,306,504-16,306,505 CC>TT mutations, −12C>T = chr3:16,306,508 C>T mutations.

a One tumor also carried additional −12C>T and another one −37G>A mutation.

b One tumor also carried −12C>T mutation.

c Out of 137 BCC tumors, for 119 matched samples from surrounding skin tissues were also tested, which did not harbor *DPH3* promoter mutations.

d One tumor also carried −12C>T and another one −13C>T mutation.

e Out of 31 SCC tumors, DNA from 19 matched surrounding skin tissues were also screened, which did not harbor *DPH3* promoter mutations.

f One tumor additionally carried −12/−13CC>TT tandem mutation.

**Figure 2:**
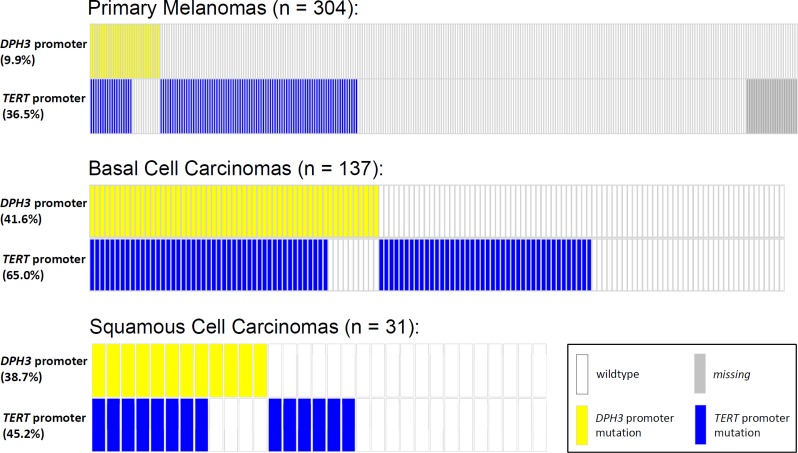
Distribution of mutations in the *TERT* and *DPH3* promoter in primary melanomas, BCCs and SCCs The mutations at the two loci occurred together more frequently than per chance with an OR of 3.0 for melanoma (95% CI 1.4 - 6.4, *P* = 0.006), OR of 3.4 for BCC (95% CI 1.5 - 7.6, *P* = 0.003) and OR of 4.3 for SCC (95% CI 0.9 - 20.2, *P* = 0.06). Two-sided *P* values and relative risk were determined by chi^2^ test.

Given the reported rate of mutations in melanoma, BCC and SCC, the frequencies of C > T base changes at −8 bp and −9 bp positions were statistically significantly higher than expected by chance (*P*<2.2×10^−16^ for each position in melanoma and BCC, binomial test; *P* = 1.7×10^−10^ and *P*<2.2×10^−16^ for −8C > T and −9C > T, respectively, in SCC). The mutations in the *DPH3* promoter tend to co-occur with *TERT* promoter mutations more frequently than expected by chance in melanoma (OR = 3.0, 95% CI 1.4 - 6.4, *P* = 0.006), BCC (OR = 3.4, 95% CI 1.5 - 7.6, *P* = 0.003) and SCC (OR = 4.3, 95% CI 0.9 - 20.2, *P* = 0.06; Figure 2). We also screened DNA from bladder tumors (*n* = 70), gliomas (*n* = 70) and squamous cell carcinoma of esophagus (*n* = 22) and none harbored *DPH3* promoter alterations.

The sites of two main mutations (−8C > T and −9C > T) were adjacent to the core binding motif 5′TTCCGG (5′CCGGAA on the reverse strand) for Ets/TCF transcription factors [1, 19]. Transcription factor binding site search of the mutated region using different algorithms resulted in identification of Ets/TCF proteins ELK1 and ELK4 sites with highest score. The mutational sites also overlapped with chromatin immunoprecipitation sequencing peaks for various transcription factors (ENCODE data), including the Ets family members ELK1, ELK4, ELF1 and GABPA (Suppl. Figure S2) [20]. The motif containing the mutations, with exception of the −8 position, is highly conserved across the mammalian lineage (Figure 1C; data from UCSC genome bowser [21]).

**Figure 3:**
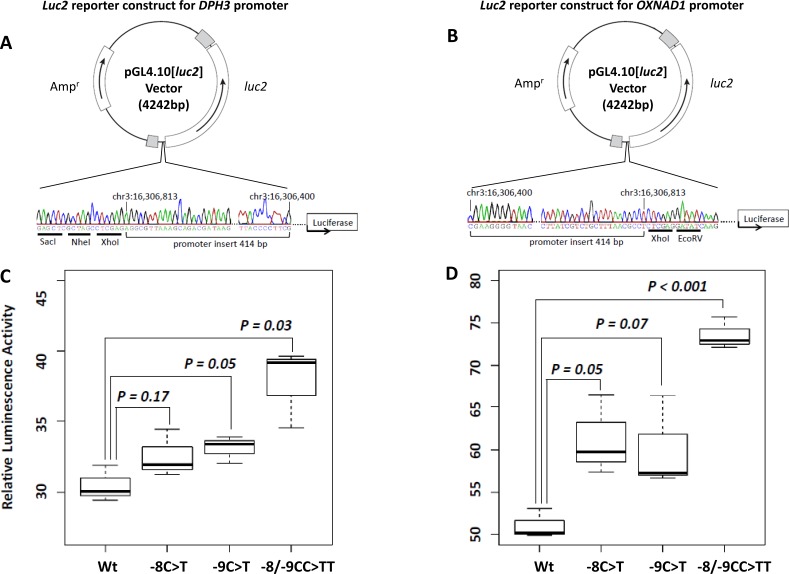
Luciferase reporter assays for the bidirectional promoter region **A.**-**B.** Luciferase reporter constructs for *DPH3* and *OXNAD1*. Promoter insert (chr3: 16,306,400-16,306,813, hg19 coordinates) was subcloned into pGL4.10[*luc2*] vector in two orientations: for *DPH3* **A.** transcribed from “-” strand and for *OXNAD1* **B.** transcribed from “+” strand. The correctness of insert orientations were confirmed by Sanger sequencing. The positive strand (containing *luc2* ATG) sequence is shown for both constructs, which corresponds to the genomic DNA positive strand for *OXNAD1* insert and negative strand in reverse orientation for *DPH3* insert. The restriction sites in the vectors are underlined. **C.**-**D.** Relative luciferase activity due to different promoter constructs. *DPH3* **C.** and *OXNAD1* **D.** promoter constructs without mutations (Wt), with −8C > T, −9C > T and −8/−9CC > TT mutations were transfected into UKRV-Mel-21 melanoma cells. The reporter activity due to the inserts with the tandem −8/−9CC > TT mutation was 1.24 and 1.44 fold higher than with wild type sequence inserts for *DPH3* and *OXNAD1*, respectively. The reporter activity in cells transfected with constructs with −8C > T or −9C > T mutations was higher than the wild type constructs but the differences were not statistically significant.

In Luciferase reporter assays carried out in two orientations, for *DPH3* and *OXNAD1*, the promoter activity due to constructs with the −8/−9CC > TT tandem mutation was statistically significantly higher than the constructs with wild type sequence (*P* = 0.03 *DPH3*; *P* = 0.001 *OXNAD1*). The constructs with the tandem mutation showed 1.24 and 1.44 fold higher promoter activity than the constructs with wild type sequence for *DPH3* and *OXNAD1*, respectively. The activity due to the promoter constructs with the −8C > T and −9C > T mutations was, though higher than the wild type constructs in both *DPH3* and *OXNAD1* orientations, but the differences were not statistically significant (Figure 3). We did not observe a statistically significant difference in the *DPH3* transcription levels based on the mutational status of the *DPH3* promoter in melanoma, melanocytic nevi and BCC tumors. In 20 tumors from SCC patients, seven with mutations and 13 without mutations, the *DPH3* transcription was lower in tumors with mutations than those without (Suppl. Figure S3). We also did not observe any effect of mutations on the transcription levels of *OXNAD1* gene in BCC (Suppl. Figure S4).

The analysis of the mutational data showed that in melanoma the *DPH3* promoter mutations associated with presence of solar lentigines at tumor sites. In BCC, *DHP3* promoter mutations associated with increased occupational sun exposure and personal history of non-melanoma skin cancers (Suppl. Table S3).

## DISCUSSION

In this communication we report frequent somatic mutations within a bidirectional promoter region of *DPH3* and *OXNAD1* genes in three major types of skin cancers. The initial discovery of the non-coding mutations within *DPH3* promoter through exome sequencing followed by a screening of 586 different skin cancer lesions showed that those promoter mutations occurred not only in 10 percent of melanoma but were also present at a frequencies of 42 percent in BCC and 39 percent in SCC. The mutations were characterized by specificity, recurrence and depicted typical UV signature as all the alterations resulted in C to T transitions at dipyrimidine sites, which was further augmented by 25 percent of all alterations in BCC and in SCC being CC > TT tandem mutations [22]. The overall frequency of the *DPH3* promoter mutations approximated that of most frequent alterations in BCC and SCC [23-25]. After *TERT* promoter mutations, this report constitutes only a second instance of highly frequent mutations being reported in human cancers within the non-coding regulatory sequences that have been called as the ‘dark matter’ of the genome [26]. However, unlike *TERT* promoter mutations, the *DPH3* promoter mutations were also present in 2 of 114 melanocytic nevi. While *TERT* promoter mutations are frequent in many cancers, we did not observe the *DPH3* mutations in three non-skin cancers that were screened in this study. Aged sun exposed normal skin has been shown to contain clones with various cancer associated mutations; however, in our study we could detect *DPH3* promoter mutations unambiguously in tumors but not in the surrounding skin [29].

Whether the detected *DPH3* promoter mutations affect transcription of adjacent genes or act as enhancers/repressors of some distant genes remains to be determined [7]. However, it may be pointed out that over-expression of Dph3 promotes the migratory ability of murine melanoma cells, whereas down regulation of Dph3 expression inhibited cellular invasion and metastasis *in vivo* [27]. The creation of Dph3 knock-out mice revealed that loss of both dph3 alleles causes a general delay in embryonic development and is embryonically lethal [28]. *DPH3* encodes a short peptide involved in electron transfer during the synthesis of eukaryotic diphthamide and forms a complex with Kti13 which is involved in both tRNA and translational elongation factor 2 (EF2) modifications [30, 31]. Diphthamide is a modified histidine residue of archaeal and eukaryotic EF2 that helps in maintenance of translational fidelity and is itself a target of translational inhibitors, diphtheria toxin and Pseudomonas exotoxin A [32, 33].

The mutations in the *DPH3* promoter were first reported in melanoma in a study based on genome wide search for mutations in the regulatory regions of the genome. The reported frequency of *DPH3* mutations in melanoma from whole genome and exome sequencing data in the Cancer Genome Atlas (TCGA) approximated 16% (6/38) and 14% (25/176), respectively [4]. The sequence element with most frequent mutations was in proximity to Ets/TCF binding motif and earlier report suggested that the immediate adjacent frequently mutated nucleotide (−9 bp position) forms the part of ELF1 binding motif [3]. Members of the Ets transcription factor family are known to act as activators as well as repressors of transcription [34-36]. In the case of the *DPH3* promoter the question of contributing transcription factors and the effect of the mutations remain elusive.

Although the luciferase reporter constructs with the −8/−9CC > TT tandem mutation showed statistically significant increased promoter activity for both *DPH3* and *OXNAD1* orientations in a melanoma cell line, we did not detect a statistically significant difference in the *DPH3* and *OXNAD1* transcription levels in melanomas and basal cell carcinomas. The expression of *DHP3* was lower in SCC tumors with mutations than those without; however, the number of tumors investigated was limited. A plausible explanation for the observed increase in promoter activity and no effect on transcription could be due to fact that the mutations alter transient transcriptional responses or temporal patterns during cell cycle progression that are not readily detectable at the whole-tumor level as discussed earlier [4].

Our study remains limited due to undetermined functionality of the mutations. However, the specificity and high recurrence of the mutations in the *DPH3* promoter, particularly in BCC and SCC and findings from an earlier report in melanoma, merits further investigations for determining the functionality and relevance of those mutations in the process of carcinogenesis [4].

## MATERIALS AND METHODS

### Patient samples

Primary tumors and corresponding blood samples from 21 melanoma patients for exome sequencing (Suppl. Table S1) and for follow up validation primary tumors from 304 melanoma patients, tumors and surrounding tissues from 137 skin BCC and 31 skin SCC patients (Suppl. Table S3) and 114 melanocytic nevi included in this study were retrieved from the biobank of the Instituto Valenciano de Oncologıa in Valencia, Spain. Of 114 melanocytic nevi, 16 were obtained from melanoma patients and 98 were from healthy individuals and of those 16 reported a family history of melanoma. Melanoma patients included in the study were classified according TNM staging system (based on the extent of the tumor (T), the extent of spread to the lymph nodes (N), and the presence of metastasis (M)) of the American Joint Committee on Cancer (AJCC) [37]. All BCC tumors were localized and did not include locally advanced tumors; SCC tumors were also localized without nodal or distant metastasis. Histological examination of the nevi included in the study showed that 11 were atypical, 84 compound, 4 congenital, 12 intradermal and 1 junctional. The nevi that carried *DPH3* promoter mutations were compound and originated from individuals that did not have personal or family history of melanoma.

Ethical approval for the study from the institutional review board of the Instituto Valenciano de Oncologıa and written informed consent from all study patients were obtained. DNA samples from 70 tumors from urothelial bladder cancer patients, 70 gliomas, and 22 tumors from patients with squamous cell carcinoma of esophagus were retrieved from the departmental collection described previously in various studies [38-40].

### Exome capture and Illumina sequencing

Exome capture was performed using Agilent SureSelect Target Enrichment System, Human All Exon V5+UTRs kit (Agilent Technologies) according to standard protocol. Capture area comprised 286754 targets from 21522 genes including untranslated regions (∼75Mb in total).

Sequencing of 21 tumor/normal pairs was carried out on Illumina Hiseq2000 with paired-end 101-nucleotide reads using protocol provided by the manufacturer. Coverage statistics was generated by Flagstat and DepthOfCoverage functions from Genome Analysis Toolkit (GATK) version 3.1-1 for the capture regions with 50 bp padding [41]. The average coverage of x68 and x64 was obtained for DNA from tumor and blood tissues, respectively (Suppl. Table S4).

### Read mapping and data preprocessing

Read pairs were mapped to the human reference genome (build hg19) using Burrows-Wheeler Aligner (BWA) version 0.7.5a mem function with default parameters [42]. BAM files were coordinate-sorted and duplicates were removed by Picard software version 1.102 (see URLs). Base quality score recalibration and local realignment around indels were performed by GATK prior to variant calling. Additional round of local realignment was performed jointly for tumor/normal pairs to avoid alignment differences in samples from one patient as suggested by GATK “best practices”. All preprocessing steps were performed for the capture regions with 50 bp padding.

### Identification of somatic single nucleotide variations (SSNVs)

Capture regions with 50 bp padding were used for variant calling to include flanking noncoding regions. Somatic single nucleotide variants were detected by Mutect algorithm [43]. The minimum base quality of 30 was required. Candidates with at least one high-quality base supporting alternate allele in the patient-matched normal sample were excluded. The C > T mutation at 16,306,505 was called in 2 samples (MEL11 and MEL12). Manual reviewing of the region using Integrative Genomics Viewer showed that sequencing reads supporting C > T mutations at 16,306,505 and/or 16,306,504 positions were present in 6 samples and out of those two samples had tandem CC > TT mutation (Suppl. Table S2) [44]. Mutect output examination revealed that the C > T mutation at 16,306,505 was originally detected in 4 samples but was filtered out in two because of the low allele frequency (“fstar_tumor_lod, possible_contamination” failure reason). The mutation at 16,306,504 was initially detected in two samples but was also filtered out.

Variant annotation was performed by ANNOVAR using RefSeq genes annotations, dbSNP (Build ID: 137, see URLs), variants from 1000 Genomes project and Catalogue of Somatic Mutations (COSMIC) version 67 [45, 46].

### Measurement of DPH3 and OXNAD1 gene expression by quantitative real-time PCR

For measurement of *DPH3* and *OXNAD1* expression, reverse transcription reaction was performed using 1 μg of RNA and oligo-dT primers using a cDNA synthesis kit (Thermo Scientific, Waltham, USA). *DPH3* and *OXNAD1* expression levels were then determined by quantitative real-time PCR using a Syber Green kit (QIAGEN). The real-time PCR was carried out in triplicates in 384-well layouts using QuantiTect primers (QIAGEN) specific for *DPH3* (QT00223083), *OXNAD1* (QT00074235) and the *GUSB* (QT00046046), a housekeeping gene used as an internal standard. *DPH3* and *OXNAD1* expression levels were calculated using *GUSB* expression as a reference and relative quantification was performed using the ΔΔCT method and log2 transformation. The expression levels were plotted using box plots and statistical differences were determined using two sided t-tests.

### URLs

Burrows-Wheeler Aligner (BWA), http://bio-bwa.sourceforge.net/; Genome Analysis Toolkit (GATK), http://www.broadinstitute.org/gatk/; Picard, http://broadinstitute.github.io/picard/; MuTect, http://www.broadinstitute.org/cancer/cga/mutect; ANNOVAR, http://www.openbioinformatics.org/annovar/; dbSNP, http://www.ncbi.nlm.nih.gov/SNP/; COSMIC, http://cancer.sanger.ac.uk/cancergenome/projects/cosmic/; Integrative Genomics Viewer (IGV), http://www.broadinstitute.org/igv/; R, http://www.R-project.org/.

## SUPPLEMENTARY MATERIAL FIGURES AND TABLES


