# Supplementary material for: Frequent DPH3 promoter mutations in skin cancers
Source: Oncotarget. 2015 Sep 21;6(34):35922–30. doi: 10.18632/oncotarget.5771 (PMC4742151; doi:10.18632/oncotarget.5771)
Supplement: Supplementary file 1 [file oncotarget-06-35922-s001.pdf]

Frequent *DPH3* promoter mutations in skin cancers

Supplementary Material

DNA and RNA extraction

DNA and RNA were extracted from fresh frozen tumor and surrounding tissues using the QIAGEN AllPrep DNA/RNA/miRNA Universal Kit. Tissues were homogenized in a Tissuelyser LT (Qiagen, Hilden, Germany) with 5 mm stainless steel beads in 600 ml RLT buffer and were further processed. Concentrations of total DNA and RNA were measured by using an ultraviolet–visible spectrophotometer (NanoDrop Technologies, Wilmington, USA) and absorption ratio at 260/280 nm was determined. RNA consistency was examined on a Bioanalyzer 2100 System (Agilent Technologies, Palo Alto, CA) using the corresponding RNA nanochips for a representative number of samples. Haematoxylin and eosin-stained sections of formalin fixed paraffin embedded (FFPE) tissues were examined for tumor content by a pathologist. Neoplastic areas were selected and dissected by tru-cut (2-3x0.6 mm) from the paraffin blocks with a manual tissue arrayer (Breecher MTA-I) to avoid stromal cells and to increase the specificity of melanoma cells to more than 95%. DNA was extracted using Qiagen DNeasy Tissue Kit (Qiagen) with additional proteinase K digestion at 55°C for 48 h.

PCR and Sanger sequencing

Determination of the mutational status of the TERT core promoter region (from position -27 to -286 from ATG start site) by PCR and Sanger sequencing was performed as described previously [1]. The mutational status of the DPH3 promoter was also determined by PCR and Sanger sequencing using one primer pair (F 5’CGAAGGGGTAACGCCCCAG 3’, R 5’GGTCCCAGACGTGACGTAGC 3’). PCR was carried out in a 10-µl volume containing 10 ng DNA, 25 mM $MgCl_2$, 0.11 mM dNTP, 5% DMSO and 0.11 mM of each primer. Temperatures

for PCR were set at 95°C for 45sec, annealing at 56°C and polymerization at 72°C for 30sec each for 35 cycles. Amplified products were purified with ExoSAP (GE Healthcare, Buckinghamshire, UK) to remove unused primer and were subjected to 35 cycles of sequencing reaction with a dideoxy terminator kit and forward and reverse primers in separate reactions (BigDyeTerminator v3.1 Cycle Sequencing Kit, Applied Biosystems, Austin, TX, USA). Sequencing reaction products were precipitated with ethanol and analysed on a capillary sequencer (AbiPrism 3130xl Genetic Analyzer). The sequencing data were analysed using Geneious Pro 5.6.5 software with reference to the sequences from the NCBI gene database, TERT (chr5: 1,295,071 - 1,295,521) and DPH3 (chr3: 16,306,256-16,306,755) (hg19 coordinates). The DPH3 promoter sequences are deposited in the GenBank database under accession numbers BankIt1784645: KP299043 - KP299143.

Cloning of DPH3 and OXNAD1 promoter region

For reporter luciferase assays a 414 bp region covering the core bidirectional promoter region (chr3: 16,306,400-16,306,813, hg19 coordinates) was amplified using genomic DNA From a single healthy individual. The amplicon was cloned into a T-overhang vector (TOPO PCR2.1, Invitrogen). The C>T (G>A on the negative strand) mutations at -8, -9 bp positions and CC>TT tandem mutation at -8/-9 bp positions from DPH3 transcription start site were generated using QuikChange Site Directed Mutagenesis kit (Agilent Technologies). A total of 4 reporter constructs carrying promoter region with wild-type (Wt) sequence, with -8T mutant, with -9T mutant and with -8/-9TT mutants were prepared for both orientations. All the plasmid constructs were sequenced by Sanger sequencing to confirm the respective mutations. For the generation of DPH3 reporter constructs the inserts from TOPO PCR2.1 were restriction digested at XhoI and BamHI sites of TOPO PCR 2.1 and subcloned into XhoI

and BglII sites of pGL4.10[luc2]. The OXNAD1 promoter inserts were restriction digested at SacI and XhoI sites and subcloned into the same sites of pGL4.10[luc2] promoter-less vector (Promega) (Figure 3).

Cell culture and Luciferase reporter assays

A patient derived melanoma cell line (UKRV-Mel-21) was a kind gift from Prof. Stefan Eichmüller of German Cancer Research Center. The cell line was derived from a Caucasian female patient with malignant melanoma. The cell line was cultured in RPMI-1640 media (Gibco) supplemented with 10% FBS (Gibco). Cells were incubated at 37°C in a humidified atmosphere with 5% CO_2. The used cell line was authenticated by short tandem repeat profiling [2]. The DNA extracted from the UKRV-Mel-21 cell line was also sequenced and found to have no endogenous mutations in DPH3 promoter.

For reporter assay, cells were seeded in 12-well plates and cotransfected with FuGene-6 (Promega), 1.5µg of reporter construct and 75 ng of pRL-actin expression plasmid for Renilla luciferase in triplicate. pGL3-control plasmid was used for determining transfection efficiency and pRL-actin as an internal control for normalization of luminescence values. pGL3-control and pRL-actin express firefly luciferase and Renilla luciferase genes under the thymidine kinase and actin promoters, respectively.

Promoter-less vector (pGL4.10[luc2]) and non-transfected cells were used as negative controls. Cells were harvested for 24 hours post transfection using 1x passive lysis buffer (Promega) and reporter expression was analyzed using the Dual-Luciferase Reporter assay system (Promega). The relative ratio of firefly luminescence to renilla luminescence was

calculated to normalize the variations across samples. Statistical differences were determined using two sided t-test and box-plots were plotted in R.

Statistical methods

We performed a binomial test to evaluate whether the frequency of C>T mutations at 16,306,504 and 16,306,505 is statistically significantly different from the average mutation rate expected per chance for the cancer types included in the study. The reported mutation rates of 11.4, 33.3 and 75.8 mutations/megabase for melanoma, squamous cell carcinoma and basal cell carcinomas respectively were used as a probability (p) of a mutation at a specific base (null hypothesis: $p=11.4x10^{-6}$; $33.3x10^{-6}$; $75.8x10^{-6}$) [3-5]. Two-sided P values with 5% significance level was calculated using R statistical software [6].

Transcription factorbinding sitespredictions

TRANSFAC databasewasusedtosearchforpredicted transcription factorbindingsiteswith three differentalgorithms including PROMO, ConSiteandrVista [7-11].

Regulation and conservation data analysis

ChIPseq data for 161 transcription factors (TFs) from the Encyclopedia of DNA Elements (ENCODE) consortium with the binding motifs from Factorbook repository was visualized using the UCSC genome browser and inspected for the DPH3 promoter region [12-14]. Other conservation and regulation data including H3K27Ac ChIP-seq peaks, which is the histone mark often found near regulatory elements, DNaseI hypersensitivity clusters,

Genome Evolutionary Rate Profiling (GERP) scores and Multiz Alignment of 100 vertebrates were also inspected using UCSC genome browser (Suppl. Figure S5).

The GERP algorithm provides a position-specific estimate of evolutionary constraint by quantifying substitute deficit across species in multiple genome alignments. It was shown previously that functional mutations tend to occur at the positions with high GERP evolutionary constraint score [15, 16]. GERP scores for the mutations positions were obtained from UCSC genome browser (http://genome.ucsc.edu/cgi-bin/hgTrackUi?g=allHg19RS_BW). According to the algorithm developers, GERP score above 2 is an indication that the position is evolutionary constraint (see the link above). GERP scores for the recurrently mutated positions “-8”, “-9”, “-12” and “-13” in the DPH3 promoter regions are 1.21, 4.29, 5.38 and 5.38 respectively, which indicates that the positions “-9”, “-12” and “-13” are highly conserved whereas position “-8” is not.

REFERENCES

1. Heidenreich B, Nagore E, Rachakonda PS, Garcia-Casado Z, Requena C, Traves V, Becker J, Soufir N, Hemminki K and Kumar R. Telomerase reverse transcriptase promoter mutations in primary cutaneous melanoma. Nature communications. 2014; 5:3401.
2. Castro F, Dirks WG, Fahnrich S, Hotz-Wagenblatt A, Pawlita M and Schmitt M. High-throughput SNP-based authentication of human cell lines. Int J Cancer. 2013; 132(2):308-314.
3. Walia V, Mu EW, Lin JC and Samuels Y. Delving into somatic variation in sporadic melanoma. Pigment cell & melanoma research. 2012; 25(2):155-170.
4. Durinck S, Ho C, Wang NJ, Liao W, Jakkula LR, Collisson EA, Pons J, Chan SW, Lam ET, Chu C, Park K, Hong SW, Hur JS, Huh N, Neuhaus IM, Yu SS, et al. Temporal dissection of tumorigenesis in primary cancers. Cancer discovery. 2011; 1(2):137-143.
5. Jayaraman SS, Rayhan DJ, Hazany S and Kolodney MS. Mutational landscape of basal cell carcinomas by whole-exome sequencing. J Invest Dermatol. 2014; 134(1):213-220.
6. Team RC. (2014). R: A Language and Environment for Statistical Computing: R Foundation for Statistical Computing).
7. Matys V, Kel-Margoulis OV, Fricke E, Liebich I, Land S, Barre-Dirrie A, Reuter I, Chekmenev D, Krull M, Hornischer K, Voss N, Stegmaier P, Lewicki-Potapov B, Saxel H, Kel AE and Wingender E. TRANSFAC and its module TRANSCompel: transcriptional gene regulation in eukaryotes. Nucleic acids research. 2006; 34(Database issue):D108-110.

8. Messeguer X, Escudero R, Farre D, Nunez O, Martinez J and Alba MM. PROMO: detection of known transcription regulatory elements using species-tailored searches. Bioinformatics (Oxford, England). 2002; 18(2):333-334.
9. Farre D, Roset R, Huerta M, Adsuara JE, Rosello L, Alba MM and Messeguer X. Identification of patterns in biological sequences at the ALGGEN server: PROMO and MALGEN. Nucleic acids research. 2003; 31(13):3651-3653.
10. Sandelin A, Wasserman WW and Lenhard B. ConSite: web-based prediction of regulatory elements using cross-species comparison. Nucleic acids research. 2004; 32(Web Server issue):W249-252.
11. Loots GG and Ovcharenko I. rVISTA 2.0: evolutionary analysis of transcription factor binding sites. Nucleic acids research. 2004; 32(Web Server issue):W217-221.
12. ENCODE project consortium. An Integrated Encyclopedia of DNA Elements in the Human Genome. Nature. 2012; 489(7414):57-74.
13. Wang J, Zhuang J, Iyer S, Lin XY, Greven MC, Kim BH, Moore J, Pierce BG, Dong X, Virgil D, Birney E, Hung JH and Weng Z. Factorbook.org: a Wiki-based database for transcription factor- binding data generated by the ENCODE consortium. Nucleic acids research. 2013; 41(Database issue):D171-176.
14. Kent WJ, Sugnet CW, Furey TS, Roskin KM, Pringle TH, Zahler AM and Haussler D. The human genome browser at UCSC. Genome research. 2002; 12(6):996-1006.
15. Cooper GM, Stone EA, Asimenos G, Green ED, Batzoglou S and Sidow A. Distribution and intensity of constraint in mammalian genomic sequence. Genome research. 2005; 15(7):901-913.
16. Cooper GM, Goode DL, Ng SB, Sidow A, Bamshad MJ, Shendure J and Nickerson DA. Single- nucleotide evolutionary constraint scores highlight disease-causing mutations. Nature methods. 2010; 7(4):250-251.

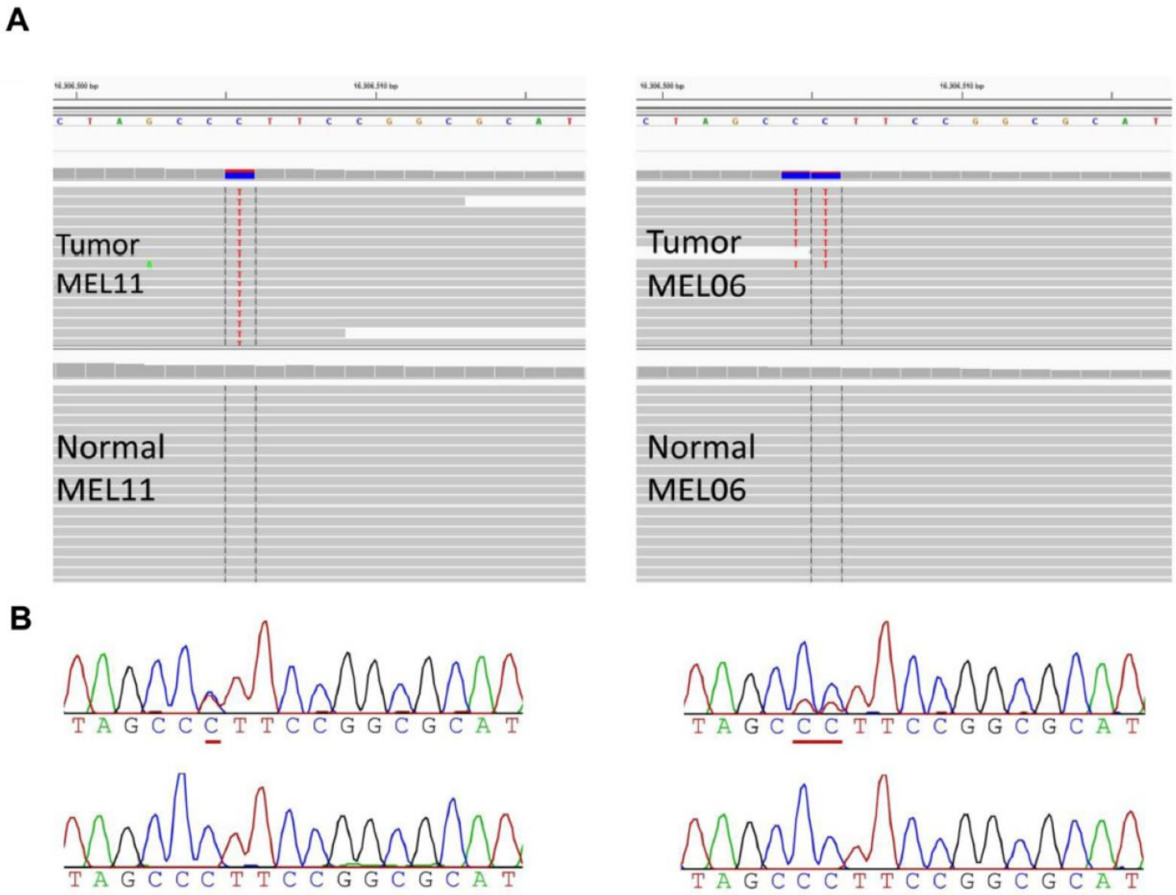

Supplementary Figure 1

Manual review of *DPH3* promoter mutations using Integrative Genomics Viewer and validation by Sanger sequencing.

Representative Integrative Genomics Viewer screenshots (**A**) and matched Sanger sequencing chromatograms (**B**) of *DPH3* promoter mutations at chr3: 16,306,505 C>T (-9C>T) and chr3: 16,306,504-16,306,505 CC>TT (-8/-9CC>TT). Average depth of coverage at the chr3: 16,306,505 was x60 for both tumor and normal samples.

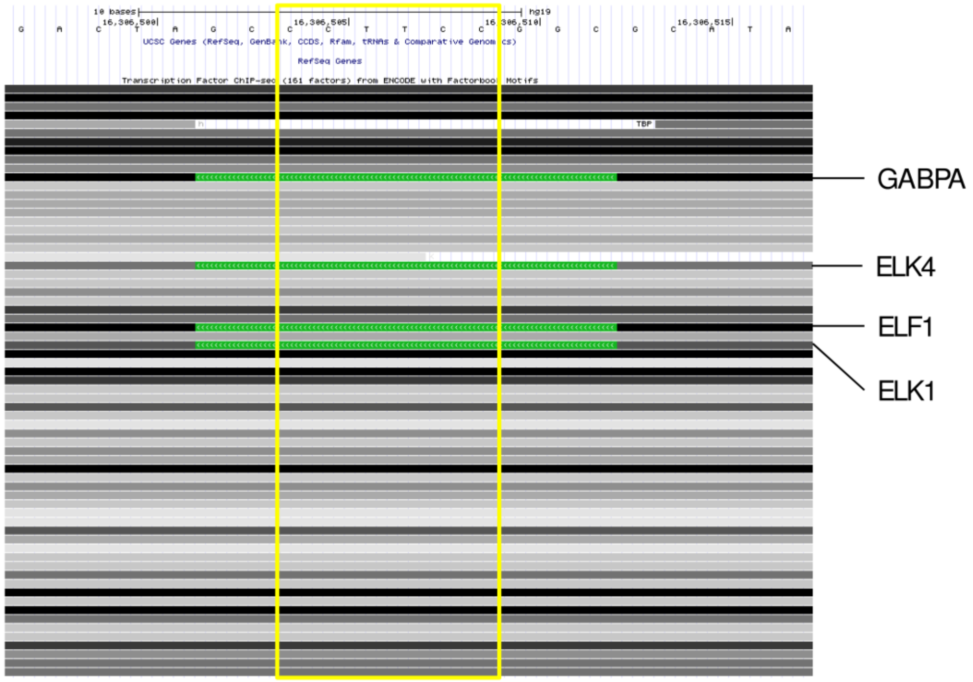

Supplementary Figure S2

ChIPseq peaks from ENCODE data in the *DPH3* promoter region. ChIPseq data for 161 transcription factors (TFs) from ENCODE, visualized using the UCSC genome browser. Each box encloses peak cluster of TF occupancy, with the darkness of the box being proportional to the maximum signal strength observed in any cell line contributing to the cluster (UCSC track description: http://ucscbrowser.genap.ca/cgi-bin/hgTables?db=hg19&hgta_group=regulation&hgta_track=wgEncodeRegTfbsClusteredV3&hgta_table=wgEncodeRegTfbsClusteredV3&hgta_doSchema=describe+table+schema) . Within a cluster, green color highlight indicates the highest scoring site of a Factorbook-identified canonical motif for the corresponding factor (Factorbook repository of ENCODE data analysis: http://www.factorbook.org/mediawiki/index.php/Welcome_to_factorbook) [12]. In particular Ets factors ELK1, ELK4, ELF1 and GABPA show high signal in the mutated region (chr3: 16,306,504-16,306,509, marked with yellow frame).

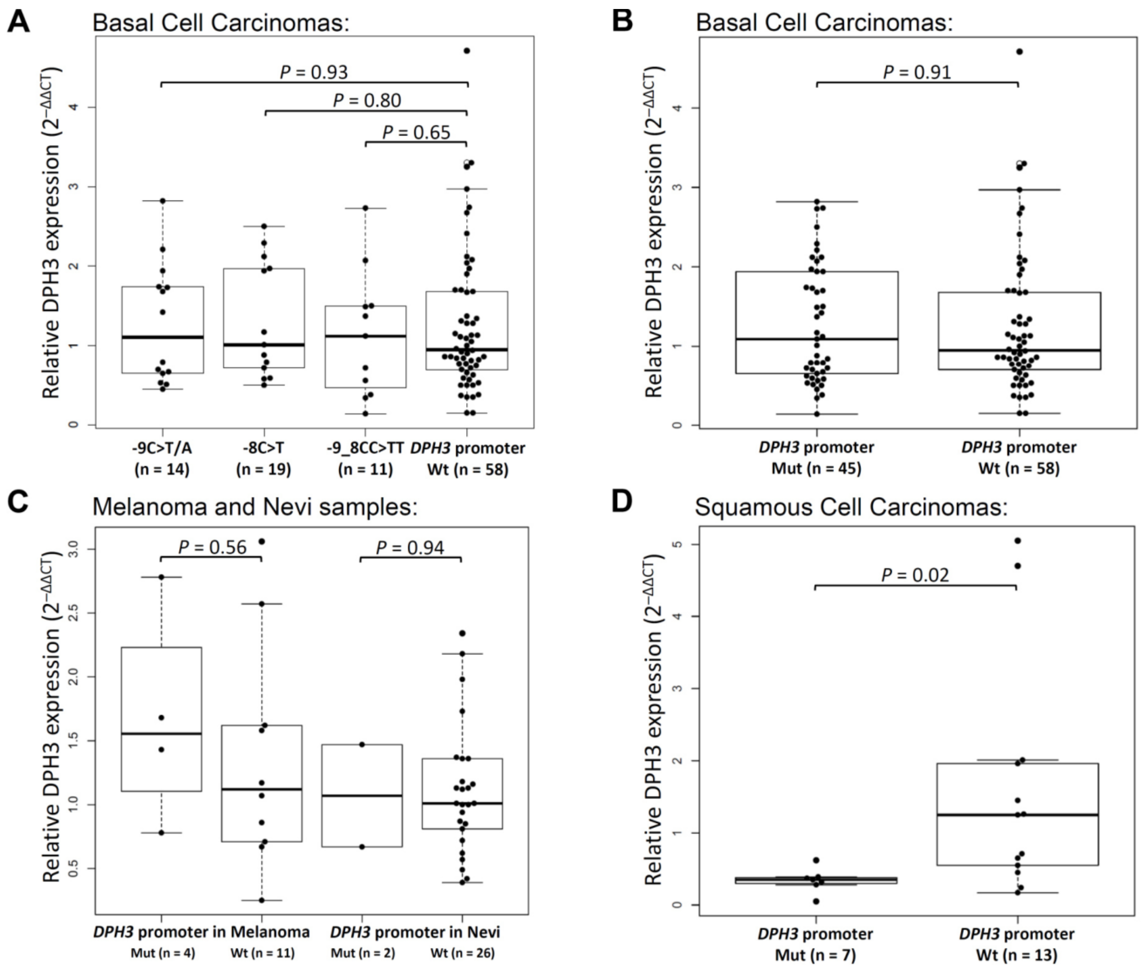

Supplementary Figure S3

***DPH3* mRNA expression in different skin lesions.** *DPH3* mRNA expression measured by quantitative real-time PCR in a different skin lesions. (**A**) Differences in the levels of expression in BCC tumors with three different mutations and without mutations (**B**) differences in expression based on presence or absence of the mutations in BCC. (**C**) Comparison of *DPH3* expression in melanoma and nevi tissues with and without mutations. (**D**) Measurement of *DPH3* expression in squamous cell carcinomas.

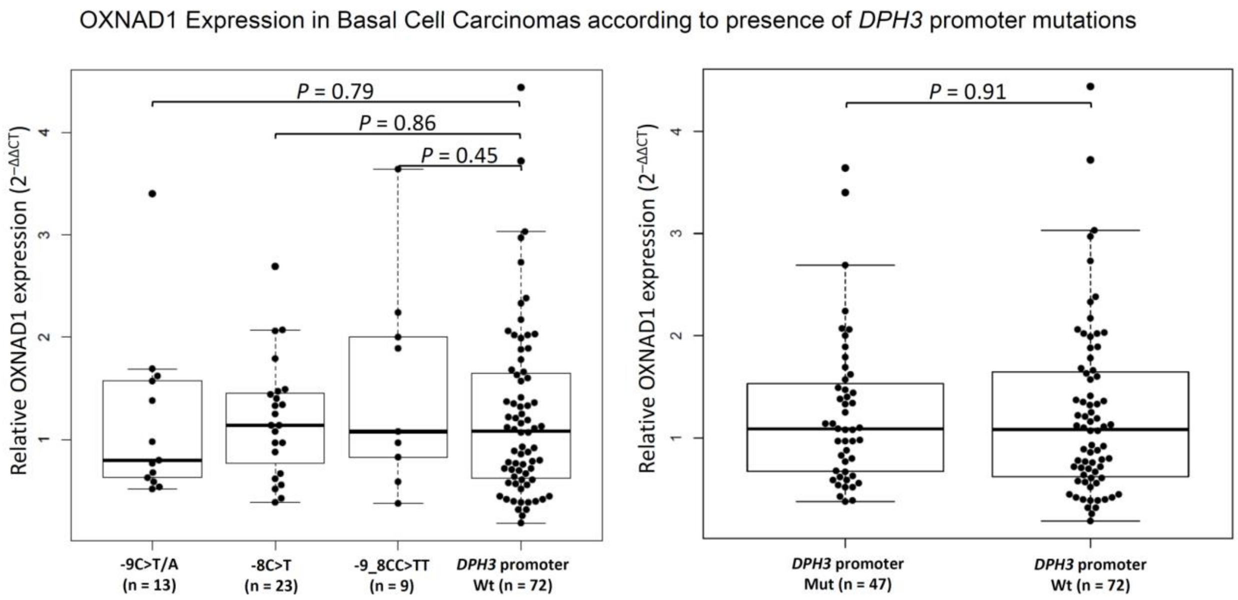

Supplementary Figure S4

***OXNAD1* mRNA expression in skin BCC.** *OXNAD1* mRNA expression measured by quantitative real-time PCR in BCC tumors with different *DPH3* mutations in the left panel. Differences in expression based on presence or absence of mutations are shown in the right panel.

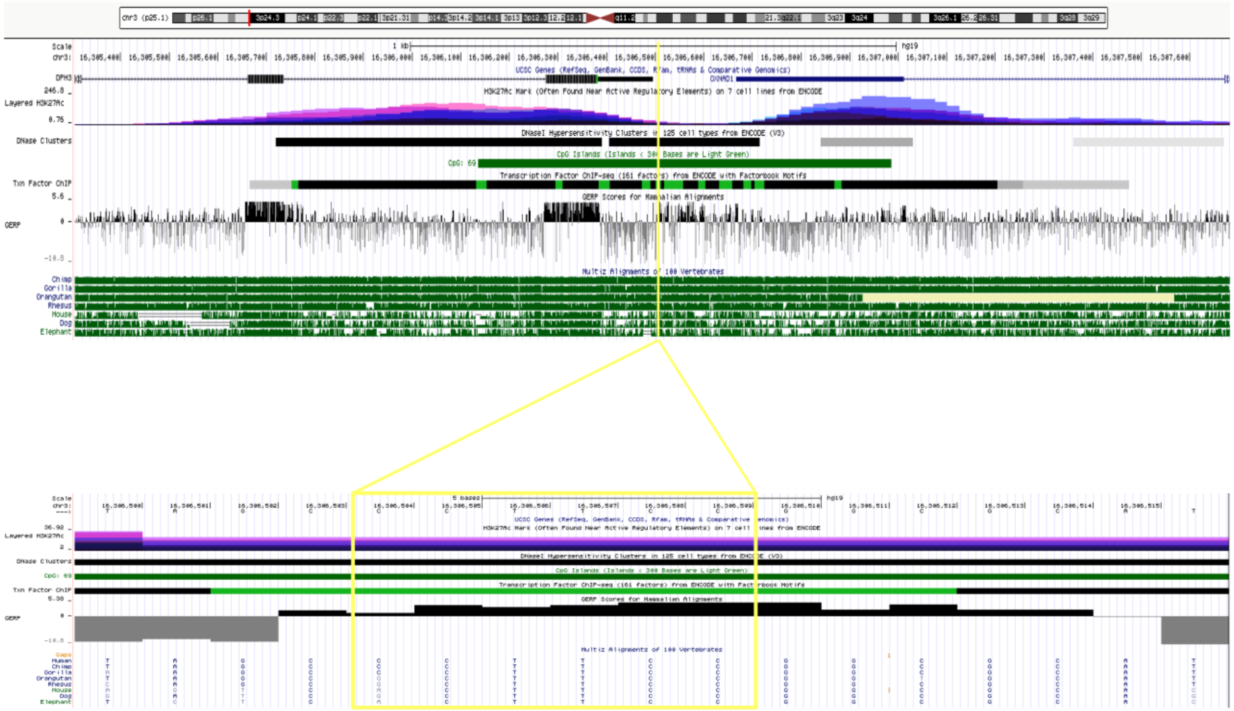

Supplementary Figure S5

Regulation and conservation data analysis in the *DPH3* promoter region. Conservation and regulation data for the *DPH3* promoter region including H3K27Ac ChIP-seq peaks (the histone mark often found near regulatory elements), DNaseI hypersensitivity clusters, CpG islands, Genome Evolutionary Rate Profiling (GERP) scores and Multiz Alignment of 100 vertebrates, visualized using UCSC genome browser. The region carries typical promoter marks and the motif containing the mutations, with exception of the -8 position, is highly conserved across mammalian lineage.

Supplementary Table S1. Clinical characteristics of 21 melanomas with whole exomes sequenced

n.a.=not available

Sample ID	Gender	Age at Diagnosis	Phototype[a]	Histological type[b]	Breslow Thickness [mm]	Clark level	Presence of Ulceration	Stage
MEL01	male	49	4	ALM	11	5	yes	3C
MEL02	female	19	2	other/unspecified	5	3	yes	3A
MEL03	female	56	4	NM	6	3	yes	3C
MEL04	female	65	2	ALM	2.9	4	yes	3B
MEL05	male	54	4	SSM	0.55	2	no	1A
MEL06	male	57	4	SSM	2.92	4	no	3A
MEL07	female	53	3	NM	12.5	5	yes	3B
MEL08	female	67	4	SSM	2.15	4	no	3A
MEL09	male	55	4	SSM	0.7	3	no	1A
MEL10	female	52	4	NM	1.85	3	no	3A
MEL11	female	82	3	LMM	2.7	4	yes	2B
MEL12	male	67	1	SSM	0.34	2	no	1A
MEL13	female	45	2	SSM	0.88	3	no	1A
MEL14	male	88	3	SSM	2.6	4	yes	2B
MEL15	female	45	2	NM	6	4	yes	3C
MEL16	male	72	3	LMM	0.25	3	no	1A
MEL17	male	50	3	SSM	2.2	4	no	2A
MEL18	female	83	4	ALM	0.6	4	no	1A
MEL19	female	65	2	SSM	1.3	4	no	1B
MEL20	female	80	2	NM	11	4	yes	3C
MEL21	female	48	2	SSM	n.a.	1	no	0

[a] Fitzpatrick phototyping scale

[b] LMM, Lentigo Maligna Melanoma; SSM, Superficial Spreading Melanoma; NM, Nodular Melanoma; ALM, Acral Lentiginous Melanoma

Supplementary Table S2. DPH3 promoter mutations in melanoma whole-exome dataset (n=21)

Sample	Sample type	-8C>T, % mutated reads[a]	-9C>T, % mutated reads
MEL11	Tumor	0.0% (0/50)	32% (16/50)[b]
MEL06	Tumor	20.0% (7/35)[b.c]	22.9% (8/35)[b,c]
MEL12	Tumor	0.0% (0/64)	10.9% (7/64)
MEL21	Tumor	7.5% (5/67)[d]	4.5% (3/67)[d]
MEL10	Tumor	0.0% (0/65)	3.1% (2/64)
MEL03	Tumor	1.4% (1/69)	0.0% (0/67)

[a]With base quality of >20 at the given position

[b]Mutations confirmed by Sanger sequencing

[c]Tandem CC>TT mutation

[d]One read supports tandem CC>TT mutation

Supplementary Table S3. Distribution and association of DPH3 promoter mutations according to patient and tumor characteristics in melanoma and non-melanoma skin cancers

Melanoma:						
		DPH3 promoter				
	All	Wt	Mut	OR	95% CI	P
All	269	241	28			
Age						
< 57	133	120	13	1	Ref.	
≥ 57	136	121	15	1.14	0.52-2.51	0.74
Gender						
Men	128	115	126	1	Ref.	
Women	141	13	15	1.05	0.48-2.31	1.00
Phototype[a]						
3 + 4 + 5	177	157	20	1	Ref.	
1 + 2	87	80	7	0.68	0.78-1.69	0.41
Not stated	5					
Eye Color						
Dark	156	142	14	1	Ref.	
Fair	108	95	13	1.39	0.62-3.08	0.42
Not stated	5					
Hair Color						
Dark	209	188	21	1	Ref.	
Fair	55	49	6	1.10	0.42-2-86	0.85
Not stated	5					
Sunburn at Area of Melanoma						
No	103	93	10	1	Ref.	
Yes, light	78	71	7	0.92	0.33-2.53	0.87
Yes, severe	68	59	9	1.42	0.54-3.70	0.47
Not stated	20					
Presence of Solar Lentigines						
No	31	30	1	1	Ref.	
Yes	226	201	25	3.73	0.49-28.56	0.17
Not stated	12					
Presence of Solar Lentigines at Melanoma Site						
No	143	137	6	1	Ref.	
Yes	118	98	20	4.66	1.81-12.03	0.00 06
Not stated	8					
Presence of Actinic Keratoses						
No	221	201	20	1	Ref.	
Yes	36	30	6	2.01	0.75-5.41	0.17
Not stated	12					
Number of Nevi						
< 50	228	205	23	1	Ref.	
≥ 51	30	27	3	0.73	0.21-2.57	0.63

Not stated	11					
Sun Exposure at Melanoma Site						
Rarely exposed	44	41	3	1	Ref.	
Infrequently exposed	189	172	17	1.35	0.38-4.83	0.64
Usually exposed	32	25	7	3.83	0.91-16.17	0.07
Not stated	4					
Histological Type[b]						
SMM	148	134	14	1	Ref.	
LMM	16	13	3	2.21	0.56-8.70	0.26
NM	67	57	10	1.68	0.70-4.00	0.24
ALM	22	22	0			
Other/unspecified	16	15	1			
Breslow Thickness						
< 2 mm	146	132	14	1	Ref.	
≥ 2mm	103	91	12	1.24	0.55-2.81	0.60
Not stated	20					
Clark Level						
1	14	13	1	1	Ref.	
2	34	31	3			
3	70	63	7			
4	124	109	15	1.24	0.55-2.78	0.61
5	18	17	1			
Not stated	9					
Presence of Ulceration						
No	190	174	16	1	Ref.	
Yes	72	61	11	1.96	0.86-4.46	0.10
Not stated	7					
Stage						
In situ	14	13	1	1	Ref.	
Localized	179	161	18			
Loco-regional disease	66	57	9	1.35	0.58-3.15	0.49
Distant metastases	4	4	0			
Not stated	6					

Basal Cell Carcinoma:

		DPH3 promoter				
	All	Wt	Mut	OR	95% CI	P
All	137	80	57			
Gender						
Women	49	30	19	1.00	Ref.	
Men	82	46	36	1.24	0.60-2.54	0.57
Not stated						
Age						
≤ 75	65	42	23	1.00	Ref.	
> 75	66	34	32	1.72	0.85-3.46	0.13
Not stated	6					

Phototype[a]						
1	8	4	4	1.00	Ref.	
2	44	22	22			
3	50	34	16	0.53	0.26-1.10	0.09
4	21	12	9			
5	1	1	0			
Not stated	13					
Nevus Count						
≤ 50	98	56	42	1.00	Ref.	
> 50	17	12	5	0.56	0.18-1.70	0.30
Not stated	22					
Sunexposure at Work						
No	72	47	25	1.00	Ref.	
Yes, < 20 years	11	7	4	1.07	0.29-4.02	0.92
Yes, ≥ 20 years	26	11	15	2.56	1.02-6.41	0.04
Not stated	28					
History of Sunburn at the Area of BCC						
No	69	40	29	1.00	Ref.	
Light	28	15	13	1.20	0.49-2.89	0.69
Severe	34	21	13	0.85	0.37-1.98	0.71
Not stated	6					
Presence of Solar Lentigines						
No	17	9	8	1.00	Ref.	
Yes, scared	52	27	25	1.04	0.35-3.12	0.94
Yes, abundant	49	32	17	2.12	0.69-6.47	0.19
Not stated	19					
Personal History of Non-Melanoma Skin Cancers						
No	28	22	6	1.00	Ref.	
Yes	60	31	29	3.43	1.22-9.66	0.02
Not stated	49					
Immunosuppression						
No	112	66	46	1.00	Ref.	
Yes	6	4	2	0.72	0.13-4.08	0.71
Not stated	19					
Squamous Cell Carcinoma:						
		DPH3 promoter				
	All	Wt	Mut	OR	95% CI	P
All	31	19	12			
Gender						
Women	10	9	1	1.00	Ref.	
Men	21	10	11	9.90	1.06-92.57	0.02
Age						
≤ 80	15	8	7	1.00	Ref.	
> 80	16	11	5	0.52	0.12-2.25	0.38
Grade						

Well differentiated (G1)	9	5	4	1.00	Ref.	
Moderately differentiated (G2)	14	7	7	1.25	0.23-6.71	0.79
Poorly differentiated (G3)	1	1	0	-	-	-
Not evaluated	7					
Sunexposure at Work						
No	10	5	5	1.00	Ref.	
Yes, < 20 years	2	1	1	1.00	0.05-20.83	1.00
Yes, ≥ 20 years	5	4	1	0.25	0.02-3.10	0.28
Not stated	14					
Personal History of Non-Melanoma Skin Cancers						
No	5	3	2	1.00	Ref.	
Yes	13	8	5	0.94	0.11-7.73	0.95
Not stated	13					
Immunosuppression						
No	18	12	6	1.00	Ref.	
Yes	2	0	2	9.61	0.40-231.43	0.07
Not stated	11					

[a] Fitzpatrick phototyping scale

[b] LMM, Lentigo Maligna Melanoma; SSM, Superficial Spreading Melanoma; NM, Nodular Melanoma; ALM, Acral Lentiginous Melanoma

Two-sided P-values were derived from χ^2-test and considered statistically significant if < 0.05.

Wt = wildtype; Mut = mutation

Supplementary Table S4. Exome Coverage Statistics

Sample ID	Sample Type	Mapped Reads	Mean Target Coverage	% Target Bases >10X	% Target Bases >20X	% Target Bases >30X
MEL01	Normal	66771722	66.0	95.1	85.3	73.9
MEL01	Tumor	67951960	67.32	95.0	85.1	73.6
MEL02	Normal	79728017	78.85	96.4	88.9	79.9
MEL02	Tumor	71554366	71.02	95.5	86.6	76.2
MEL03	Normal	68190257	67.49	94.6	84.8	73.5
MEL03	Tumor	75726265	74.91	96.1	87.9	78.1
MEL04	Normal	65981824	65.29	94.9	85.0	73.6
MEL04	Tumor	68544562	67.8	95.4	86.2	75.3
MEL05	Normal	66527918	65.94	95.0	85.1	73.7
MEL05	Tumor	58810095	58.43	93.5	81.7	68.6
MEL06	Normal	61818406	61.03	94.4	83.6	71.2
MEL06	Tumor	54192258	53.8	92.4	78.9	64.4
MEL07	Normal	71158548	70.28	95.7	87.1	76.7
MEL07	Tumor	77132859	76.61	96.0	88.0	78.5
MEL08	Normal	62765321	62.17	94.4	83.8	71.5

MEL08	Tumor	57487282	57.16	92.9	80.4	66.7
MEL09	Normal	63650331	63.14	94.3	83.8	71.9
MEL09	Tumor	77048140	76.49	95.9	87.7	78.1
MEL10	Normal	71535737	70.92	95.5	86.6	76
MEL10	Tumor	73033097	72.26	95.8	87.4	77.3
MEL11	Normal	64197689	63.78	94.4	84.0	72.2
MEL11	Tumor	59805761	59.26	93.6	81.8	68.7
MEL12	Normal	68234033	67.88	94.8	85.0	74.0
MEL12	Tumor	77268209	76.77	95.9	87.7	78.1
MEL13	Normal	64695528	64.03	94.9	84.9	73.2
MEL13	Tumor	62483332	62.01	94.2	83.2	70.7
MEL14	Normal	59131392	58.6	93.8	82.2	69.2
MEL14	Tumor	57912646	57.51	92.6	79.9	66.3
MEL15	Normal	71135285	70.65	95.3	86.3	75.9
MEL15	Tumor	62352593	61.88	94.3	83.5	71.2
MEL16	Normal	56944380	56.63	93.0	80.6	67.0
MEL16	Tumor	56870075	56.51	93.1	80.6	67.0

MEL17	Normal	55418712	55.12	92.6	79.8	66.0
MEL17	Tumor	74140178	73.49	95.7	87.0	76.7
MEL18	Normal	57791525	57.45	93.3	81.4	68.0
MEL18	Tumor	76277438	75.54	96.1	88.0	78.1
MEL19	Normal	51469457	51.15	92.0	78.2	63.2
MEL19	Tumor	66706462	66.28	94.8	84.8	73.3
MEL20	Normal	65775929	65.36	94.7	84.8	73.4
MEL20	Tumor	80639722	80.12	96.3	88.7	79.6
MEL21	Normal	61992563	61.46	94.3	83.6	71.3
MEL21	Tumor	79129801	78.53	96.2	88.5	79.3
